# Influence of Processing Conditions on the Flavor Profiles of Mulberry (*Morus alba* Linn) Fruits Using Instrumental Flavor Analysis and Descriptive Sensory Analysis

**DOI:** 10.3390/foods9050581

**Published:** 2020-05-05

**Authors:** In-Seo Hwang, Mina K. Kim

**Affiliations:** Department of Food Science and Human Nutrition and Fermented Food Research Center, Jeonbuk National University, 567 Baekjedaero, Deokjin-gu, Jeonju-si, Jeonbuk 54896, Korea; his0753@naver.com

**Keywords:** mulberry fruit, volatile compounds, gas chromatography–mass spectrometry (GC-MS)., purge and trap (P&T), distilled under reduced pressure (DRP), liquid–liquid extraction (LLE), sensory analysis, descriptive analysis, heat treatment

## Abstract

The purpose of this study was to identify the influence of drying method on flavor profiles of mulberry fruit using purge and trap (P&T) flavor extraction followed by gas chromatography–mass spectrometry (GC-MS) and descriptive sensory analysis using a highly trained sensory panel. Mulberry fruit samples were prepared at different temperatures (−20, 0, 50, and 60 °C). The results showed that more diverse volatile compound profiles were produced overall and had increased levels of benzaldehyde, nonanal, and 3,3-dimethylhexane in Sample 3 and 4, which were dried at higher temperature (50 °C and 60 °C). The mulberry fruit samples that received heat treatment had higher grape juice, raisin, and sour aromatics, while samples that did not received heat treatment were characterized as having cucumber, green/grassy, and sweet aromatics.

## 1. Introduction

Mulberry (*Morus alba* Linn) fruit, also known as *Odie* in Korean, is a common fruit consumed in East Asian countries such as Korea, China, Japan, and Thailand [1]. Due to the presence of polyphenols, flavonoids, anthocyanins, essential amino acids, and vitamins [2], mulberry fruit extracts are well-known for their health-promoting effects, including antioxidant activities, antiradical capacities, antidiabetic properties, hypolipidemic effects [3], and neuroprotective effects [3]. Consumers of mulberries in Korea are aware of the health-promoting effects associated with this fruit. Jeollabuk-do, located in Korea’s southwest region, is famous for cultivating mulberries. Due to the fragility and perishability of mulberry fruit, these fruits are distributed in the market as mulberry extracts, jams, juice, and mulberry flavor-added alcoholic beverages [4]. For the development of value-added mulberry-processed products, understanding the flavor of mulberry is a necessity because many studies have reported that flavor is the key driver for consumers to purchase food products such as orange juice [5], Greek yogurt [6], and mild cheddar cheese [7].

To date, only a limited number of studies have been conducted on the flavor analysis of mulberry. Most flavor analyses of mulberry have focused on mulberry wines, such as investigating the impact of yeast on flavor development [8]. Kwaw et al. [9] reported the influence of lactic fermentation to the flavor of mulberry juices, and study of mulberry leaf tea has also been reported [10]. A few studies have been conducted to analyze the flavor profiles of fresh fruits. Elmacı and Altuğ [11] reported an instrumental and sensory flavor analysis of *Morus nigra* fruits from different cultivars and reported that ethyl linolenate could be a key compound in determining its flavor acceptability. Chen et al. [12] reported an instrumental flavor analysis of *Morus atropurpurea* fruits in different cultivars and found that glycosidically bound volatiles from fruit can be applied for aroma enhancement or modification.

As mentioned above, previous research has focused on the flavor components of mulberry wine, tea, or juices. However, the flavor of mulberry fruit before processing and impact of each flavor component analyzed from various studies of mulberry fruits are still unknown. Therefore, the objective of this study was to comprehensively understand the flavor of mulberry fruits prepared at different temperatures with instrumental flavor analysis using purge and trap (P&T) flavor extraction followed by gas chromatography–mass spectrometry (GC-MS) and descriptive sensory analysis using a highly trained sensory panel. Correlation analyses between instrumental flavor analysis and sensory evaluation results were also conducted to determine the sensory characteristics associated with volatile aromatic compounds isolated from mulberry fruits.

## 2. Materials and Methods

### 2.1. Sample Preparation

The mulberries used for this study were purchased from a local producer (chambbong-odie, Bu-an, Jeollabuk-do, South Korea). Upon receipt, the mulberries were prepared accordingly. Four different drying temperatures were selected: −20 °C (Sample 1), 0 °C (Sample 2), 50 °C (Sample 3), and 60 °C (Sample 4). Samples 3 and 4 were dried using a food dehydrator (LD-918BT, Lequip, Hwaseong, South Korea) for 8 h. The drying temperatures for this study were selected because they represent the typical distribution scenario of mulberries in Korea. For example, −20 and 0 °C represent the typical storage conditions of mulberry fruits upon harvest, and 50 and 60 °C represent the typical drying temperatures of commercially-available dried mulberry fruits. All samples were stored in a freezer after preparation except those that required storage in a refrigerator.

### 2.2. Flavor Extraction Using Distillation under Reduced Pressure

Once dried, flavor constitutions of mulberry samples were extracted using the distillation under reduced pressure method. In this step, deionized water was used as a solvent; the ratio was 500 g to 3 L of water for Samples 1 and 2 and 500 g to 5 L of water for Samples 3 and 4. Differing amounts of solvent water were unavoidable because of the density differences between samples, and the amounts were determined from the author’s preliminary study [13]. After dissolving, all samples were heated up to 56 °C for 2 h using a stirring mantle (MS-ES303, MTOPO, Seoul, South Korea) with addition of 50 g of glass boiling stones (Sigmund^®^, Warmensteinach, Germany) during extraction to avoid overflowing. Constant agitation at 400 rpm was proceeded during extraction While pressure was reduced down to 0.017 MPa to lower the boiling point. Once distillation was completed, samples were sent to a circulator (CCA-1112, EYELA, Shanghai, China) with the temperature set at 10 °C.

This was followed by a continuous LLE to collect flavor constituents from the extracts. Dichloromethane (DCM; J. T. Baker, Radnor, PA, USA) was used as a solvent for LLE; 100 mL of DCM was added to a round bottom flask, and 30–50 mL of DCM was added into the liquid–liquid extractor to avoid overflow into the flask. Then, 300 mL of distilled samples and deionized water were added if the volume of the sample did not reach the top. The circulator was kept at 10 °C. After all pieces of the apparatus were connected, the flask was heated up in a hot water bath at 55 °C for 6 h. After extraction, each sample was stored in the freezer for 1 day, with some sodium sulfate added for dehumidification.

### 2.3. Concentration of Volatile Components

Extracted samples were initially filtrated to eliminate sodium carbonate in the samples. Each sample was connected to a rotary vacuum evaporator (N-1300, EYELA, Tokyo, Japan) set at 130 rpm in a heated water bath kept at 50 °C to become about 1 mL. An aspirator (A-1000S, EYELA, Tokyo, Japan) was also connected to the evaporator to lower the pressure for easier evaporation of DCM. Evaporated samples were heated up in a dry block bath at 37 °C with liquid nitrogen to evaporate excess DCM. This resulted in a final volume of 0.4−0.5 mL.

### 2.4. Volatile Compound Analysis

A P&T sample using the AQ-200 liquid sampler (Japan Analytical Industry, Tokyo, Japan) was applied for volatile flavor analysis. Briefly, 100 μL of each extracted sample was volatilized at 60 °C for 30 min, and flavor compounds purged from the samples were absorbed onto Tenax GR fibers (Japan Analytical Industry). Fibers were desorbed at 280 °C for 30 min and were trapped in the curio pointer (−40 °C).

After P&T, each sample underwent pyrolysis at 280 °C prior to injection into the GC column. The conditions for this step were as follows. The transfer line temperature was set to 280 °C, the needle heater was set to 280 °C, and the cold-trap heater was set to 200 °C with the head press at 86 MPa. The column flow was set at 1.0 mL/min with a split ratio of 1/100. GC/MS QP 2010 plus (Shimadzu, Kyoto, Japan) was used for flavor compounds analysis. The DB-624 column (30 m × 0.25 mm × 1.40 μm; Agilent Technologies, Wilmington, DE, USA) was used. The oven temperature was programmed with the following conditions: 40 °C for 3 min hold, 10 mL/min up to 260 °C, 5 min hold at 260 °C. The ion source was 200 °C, the transfer line was 250 °C, and the electromagnetic voltage was set at 70 eV.

The identification of volatile compounds was conducted by comparing retention indices in the NIST database (Wiley 9.1v). Peak area ratio (PAR) of each identified volatile compound was recorded for quantification. PAR was calculated as

(1) $$\mathrm{PAR}\;\left(\%\right)=\frac{\mathrm{GC}\;\mathrm{Peak}\;\mathrm{Area}}{\mathrm{Total}\;\mathrm{GC}\;\mathrm{Peak}\;\mathrm{Area}}\times 100$$

### 2.5. Descriptive Sensory Analysis of Mulberry Fruits

Descriptive sensory analysis of mulberry fruits was conducted using a highly trained panel consisting of four females and one male ranging in age from 23 to 41 years old. Each panelist had over 150 h of experience in sensory evaluation for various food products using the Spectrum^TM^ method [14]. The 10 g mulberry fruit samples were served in 56-mL plastic cups with plastic lids (Sujeong packaging, Seoul, Republic of Korea) labeled with three-digit randomized numbers. Samples were prepared 1 h prior to sensory evaluation in order to temper the sample temperature to room temperature. Panelists were asked to evaluate the aroma perceived from the headspace of the sample cup; therefore, no tasting was involved.

Prior to actual evaluation, two 2 h training session was conducted to calibrate the use of universal scale in the Spectrum^TM^ method. Upon completion of the training session, panelists generated the sensory lexicon to describe the aroma characteristics of mulberry fruits, and a reference of sensory terms was provided to assist panelists with this step. Aroma intensity measurement using a 15-pt universal scale was conducted for each sensory lexicon. During the evaluation, the panel rested for at least 2 min between samples to minimize fatigue and carry-over effects. Paper ballots were used for data collection.

### 2.6. Statistical Analysis

Data analysis was conducted using XLSTAT (v.2018, Addinsoft, Paris, France). One-way analysis of variance was followed by Duncan’s multiple range test for determination of sample differences at α = 0.05 level. Principal component analysis (PCA) was conducted to determine where each sample was located in the sensory and flavor characteristics map and to correlate the sensory characteristics to volatile flavor analysis results.

## 3. Results and Discussion

### 3.1. Instrumental Analysis of Volatile Flavor Compounds in Mulberry Fruit

The flavor compounds identified from four mulberry samples and the PAR of each identified volatile compound are shown in Table 1. A total of 30 volatile aromatic compounds were identified in mulberry fruits treated at different temperatures. Among them, 12 compounds were present in all samples: ethyl acetate, chloroform, 1,3-cyclohexadiene, vinylcyclobutane, 2,4-dimethylheptane, 4-methyloctane, cyclopentanecarboxaldehyde, cyclohexene oxide, 2-cyclohexen-1-one, benzaldehyde, phenol, and nonadecane.

These compounds were present in different levels in the four mulberry samples. In particular, the level of ethyl acetate was significantly higher in Sample 3 (1.509) than the other samples (*p* < 0.05). This compound is known to have fruity aroma characteristics [15], and this result suggests the loss of fruity aromatics in samples heated at 60 °C. Vinylcyclobutane showed the highest PAR (51.694−63.581) in all samples in comparison to the PAR of other volatile compounds, and no significant differences in vinylcyclobutane levels were observed between samples (*p* < 0.05). Vinylcyclobutane itself does not produce its own aroma characteristics. However, this compound can be thermally rearranged into other volatile aromatic compounds such as cyclohexene and butadiene as well as ethylene at high temperatures such as 300 °C [16]. Mulberry fruits were treated at −20, 0, 50, and 60 °C prior to volatile flavor analysis; therefore, thermal rearrangements of vinylcyclobutane did not occur during the processing condition. The PAR of 2-cyclohexen-1-one was the second-highest in the result while vinylcyclobutane showed the highest PAR, and 2-cyclohexen-1-one has a fresh air and sweet aroma [17]. Thermal degradation of 2-cyclohexen-1-one could form phenol [18]. The PAR of phenol, which has a plastic smell [19], was the highest in Sample 3 (2.399; *p* < 0.05).

A significant correlation was found between the PAR of benzaldehyde and drying temperature, with PARs of 0.262 (Sample 1), 0.432 (Sample 2), 1.106 (Sample 3), and 1.222 (Sample 4) (*p* < 0.05). This compound is known for its almond-like aroma characteristics [20], and its formation results from the reduction of esters and/or oxidation of alcohols. The heating treatment in Samples 3 and 4 may have influenced the higher proportion of benzaldehyde in those two samples compared to the non-heat-treated samples.

In addition to the above-mentioned compounds found in all four samples, several compounds were identified specifically in one or two samples. For example, hexanal was identified in Samples 2 and 3, and the PAR was significantly higher in Sample 2 (1.010 vs. 0.361; *p* < 0.05). This compound is characterized by green- and apple-like aroma characteristics [21]. Nonanal was only found in heat-treated samples, with a PAR of 0.362 in Sample 3 and 0.425 in Sample 4, with no significant difference between samples. This compound is known for its fatty, green, and sweet aroma characteristics [22].

At least half of the samples contained two cyclic alcohols, cyclohexanol (in Samples 1–3) and 2-cyclohexen-1-ol (in Samples 1 and 2). Cyclohexanol has aromatic camphor-like characteristics [23], while 2-cyclohexen-1-ol has caramelized, phenolic, and floral aroma characteristics [24]. These two cyclic alcohols are formed through a Brønsted acid–base reaction with the addition of oxygen, the major by-products of which are cyclic ketones such as cyclohexanone and 2-cyclohexen-1-one. Further reactions can form phenol and 2-cyclohexene-1,4-dione [25]. While these two cyclic alcohol compounds (cyclohexanol and 2-cyclohexen-1-ol) were detected in samples treated at lower temperatures (Samples 1, 2, and/or 3), cyclic ketone compounds (2-cyclohexen-1-one) were found in all samples, including Sample 4. The presence of 2-cyclohexen-1-one and the absence of cyclic alcohol compounds in Sample 4 may be attributed to the Brønsted acid–base reaction during the heat-treated drying process at 60 °C. Similar to 2-cyclohexen-1-one, 2-methylene cyclopentanol, nonanal, and 3,3-dimethylhexane were only detected in heat-treated samples.

### 3.2. Descriptive Sensory Analysis Results

Table 2 presents the results of the descriptive sensory analysis of mulberry fruits treated at different temperatures. A total of eight sensory attributes were generated to describe the sensory aroma characteristics of mulberry fruits: cucumber, grassy, sweet aromatics, raisin, sour aromatics, grape juice, dried seafood storage, and medicinal. The definition and sensory reference of each sensory term is presented in Table 2.

Elmacı and Altuğ [11] reported descriptive sensory analysis results for the aroma attributes of *Morus nigra*, which included fruity, acid, musky, leafy, and woody-fresh. Calín-Sánchez et al. [30] conducted a study in Spain to describe the sensory analysis of fresh mulberries (*Morus nigra* and *Morus alba*), which were described as having mulberry, ripe fruit, green fruit, fruity, and floral aroma characteristics. Likewise, a descriptive sensory analysis of different species of mulberry musts (*Morus nigra*, *macroura*, and *alba*) reported that the fruit has mellow, fruity, acidic, green, sweet, and sulfur aroma characteristics [31].

Significant differences in the intensities of all aroma attributes were observed between the four mulberry samples (*p* < 0.05). The differences in aroma characteristics were mainly attributed to heat treatment, with non-heat-treated samples (Samples 1 and 2) having more intense cucumber, green, and sweet aromatics than the samples treated with heat. The mean intensity of cucumber aromatics in Samples 1 and 2 were 2.50 and 1.55, respectively, and the aroma intensity of cucumber and green/grassy attributes in Sample 1 were significantly higher than in Sample 2 (*p* < 0.05). There was no significant difference in the intensities of sweet aromatics between Samples 1 and 2 (0.87 and 0.80), though these were still significantly higher than the intensity in Sample 3. In previous study, the attribute cucumber was used for the sensory lexicon of Charentais melons [32]. The study by Hongsoongnern and Chambers IV [33] stated that the attribute cucumber can be positioned as a subordinate or detailed concept of the green/grassy attribute.

The aroma attributes raisin, sour aromatics, grape juice, and dried seafood storage were only perceived in samples that were heat treated (Samples 3 and 4). The intensities of raisin and dried seafood storage attributes were significantly higher in Sample 3 than Sample 4. The intensity of raisin was 2.00 in Sample 3 and 1.48 in Sample 4. The intensity of dried seafood storage was 0.70 in Sample 3 and 0.15 in Sample 4. The attribute grape juice contains bitter and musty aroma characteristics, and Chambers et al. [34] also reported bitter and musty characteristics in brewed coffee aromatics. The attributes grape and dark-fruity, which are similar aroma characteristics as in raisins, were also found in the descriptive sensory analysis results for pomegranate juice [35]. Interestingly, heat-treated pomegranate juice (dehydrated at 57 °C for 10 h) showed the highest score for dark-fruity aromatics, which is similar to the findings of this study.

It is worth noting that a medicinal aroma was exclusively noted in Sample 2, which was stored at 0 °C. A medicinal aroma was noted in the descriptive sensory analysis of orange juice [5]. According to Kim et al. [36], a medicinal flavor seems to have a higher correlation with a bitter taste rather than a specific volatile compound. This study focused on aroma perception, meaning that only orthonasal analysis was conducted. Therefore, the bitter taste of mulberry fruit samples was not evaluated, and a correlation between medicinal aromatics and a bitter flavor in mulberry fruits is still unknown.

### 3.3. PCA of Mulberry Fruits Treated at Different Temperatures

**Figure 1:**
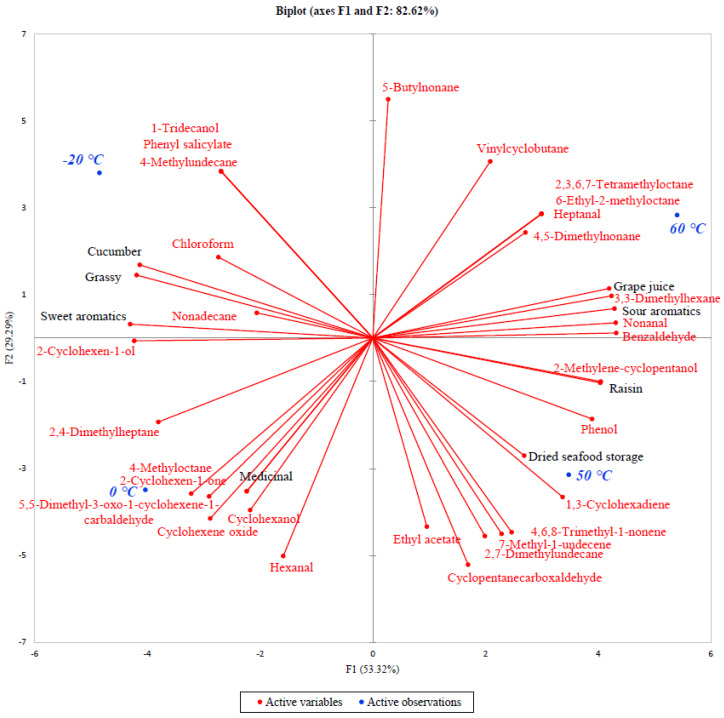
Principal component analysis biplot on volatile flavor analysis and descriptive sensory analysis analyses of mulberry fruits treated in different temperatures.

A PCA biplot of the mulberry fruits treated at different temperatures can be found in Figure 1, which shows where each mulberry fruit sample is located on a sensory and instrumental flavor analysis map. APCA biplot explains about 82.62% of the total variability of the dataset; horizontal axis (F1) explains 53.32% of total variability and vertical axis (F2) explains the remaining 29.29%. Samples dried at different temperatures were clustered along the horizontal axis; samples that received heat treatment (Samples 3 and 4) were located in positive F1, while those that did not receive heat treatment (Samples 1 and 2) were located in negative F1.

Samples located in negative F1 were characterized as having cucumber, grassy, and sweet aromatics, which show high correlations with 2-cyclohexen-1-ol, nonadecane, 4-methylundecane, and 1-tridecanol. Samples 1 and 2 showed slightly different sensory and volatile profiles in that Sample 1 showed more correlation towards the above-mentioned aroma characteristics (cucumber, grassy, and sweet aromatics), while Sample 2 was characterized as having a medicinal aroma. The flavor compounds that have shown more correlation to medicinal aromatics include 4-methyloctane, 2-cyclohexen-1-one, 5,5-dimethyl-3-oxo-1-cyclohexene-1-carbaldehyde, cyclohexene oxide, cyclohexanol, and hexanal. Some of these compounds may have influenced the medicinal aromatics of Sample 2.

Samples located in positive F1 (Samples 3 and 4) shared similar sensory and instrumental flavor profiles, with slight differences between the two samples. Overall, samples in positive F1 were characterized as having grape juice, sour, and raisin aromatics, and 3,3- dimethylhexane, nonanal, benzaldehyde, and 2-methylene-cyclopentanol were highly correlated with these sensory characteristics. Sample 3 was specifically characterized as having dried seafood storage aromatics, which could be an off-putting flavor. Sakakibara et al. [37] reported that a dried fish odor can be derived from phenols as a result of lipid oxidation. The presence of lipid oxidation by-products, such as phenol, cyclopentanecarboxaldehyde, and 2,7-dimethylundecane, could have resulted in the dried seafood storage aroma characteristic in Sample 3.

## 4. Conclusions

In this study, instrumental flavor analysis and descriptive sensory analysis of mulberry fruits treated at four different temperatures were documented. Heated drying of mulberry fruit increased the levels of benzaldehyde, nonanal, and 3,3-dimethylhexane. The samples that received heat treatment had higher grape juice, raisin, and sour aromatics, while the samples that did not receive heat treatment were characterized as having cucumber, green/grassy, and sweet aromatics. The findings from this work can assist mulberry producers with the practical development of value-added food products using mulberries by providing the key flavor compounds responsible for their desirable characteristics.

## Figures and Tables

**Table 1:** Instrumental analysis results on volatile compounds of four mulberries treated in different temperatures.

NO.	Compound Names	Odor Description	RT	ID	PAR				Ref.
					Sample 1	Sample 2	Sample 3	Sample 4	
1	Ethyl acetate	Fruity	6.005	RI, MS	0.619 ^c^	0.990 ^b^	1.509 ^a^	0.589 ^c^	[15]
2	Chloroform	Floral	6.311	RI, MS	0.855	1.115	0.703	0.864	[26]
3	1,3-Cyclohexadiene	-	7.059	RI, MS	1.823	2.542	2.545	2.246	-
4	Vinylcyclobutane	-	7.196	RI, MS	51.694	63.581	58.327	62.499	-
5	2,4-Dimethylheptane	-	10.459	RI, MS	1.266 ^b^	1.853 ^a^	1.342 ^b^	1.198 ^b^	-
6	Hexanal	Green-like, apple-like	10.839	RI, MS	-	1.010 ^a^	0.361 ^b^	-	[21]
7	4-Methyloctane	-	11.423	RI, MS	0.997 b	1.494 ^a^	1.188 ^ab^	1.007 ^b^	-
8	Cyclopentanecarboxaldehyde	-	11.955	RI, MS	0.654	0.911	0.900	0.753	-
9	Cyclohexene oxide	-	12.142	RI, MS	2.108 ^b^	3.128 ^a^	2.600 ^ab^	2.197 ^b^	-
10	Cyclohexanol	Camphor-like	13.061	RI, MS	0.230 ^b^	0.379 ^a^	0.403 ^a^	-	[23]
11	Heptanal	Green, sweet	13.172	RI, MS	-	-	-	0.453 ^a^	[27]
12	2-Methylene-cyclopentanol	-	13.177	RI, MS	-	-	2.641 ^a^	1.820 ^b^	-
13	2-Cyclohexen-1-ol	Caramelized, phenolic, floral	13.182	RI, MS	2.047 ^b^	3.025 ^a^	-	-	[24]
14	2-Cyclohexen-1-one	Fresh air, sweet	14.419	RI, MS	4.792 ^ab^	6.622 ^a^	6.042 ^ab^	4.341 ^b^	[17]
15	Benzaldehyde	Almond-like	14.832	RI, MS	0.262 ^c^	0.432 ^b^	1.106 ^a^	1.222 ^a^	[20]
16	2,7-Dimethylundecane	-	15.538	RI, MS	-	5.136 ^a^	4.299 ^ab^	3.431 ^b^	-
17	7-Methyl-1-undecene	-	15.964	RI, MS	-	0.533 ^a^	0.499 ^a^	0.384 ^b^	-
18	Phenol	Phenolic	16.106	RI, MS	1.537 ^b^	2.084 ^ab^	2.399 ^a^	2.049 ^ab^	[28]
19	6-Ethyl-2-methyloctane	-	16.169	RI, MS	-	-	-	0.609 ^a^	-
20	4,5-Dimethylnonane	-	16.310	RI, MS	4.813 ^a^	-	7.071 ^a^	5.506 ^a^	-
21	4-Methylundecane	-	16.364	RI, MS	0.785 ^a^	-	-	-	-
22	Nonanal	Fatty, green, sweet	17.055	RI, MS	-	-	0.362 ^a^	0.425 ^a^	[22]
23	Nonadecane	-	19.582	RI, MS	3.055	3.311	3.738	2.522	-
24	1-Tridecanol	Musty	19.766	RI, MS	0.506 ^a^	-	-	-	[29]
25	5-Butylnonane	-	19.911	RI, MS	1.770 ^b^	0.731 ^c^	-	2.775 ^a^	-
26	4,6,8-Trimethyl-1-nonene	-	20.115	RI, MS	-	0.565 ^a^	0.575 ^a^	0.425 ^b^	-
27	3,3-Dimethylhexane	-	20.242	RI, MS	-	-	1.067 ^b^	2.377 ^a^	-
28	2,3,6,7-Tetramethyloctane	-	20.420	RI, MS	-	-	-	0.308 ^a^	-
29	5,5-Dimethyl-3-oxo-1-cyclohexene-1-carbaldehyde	-	20.963	RI, MS	-	0.558 ^a^	-	-	-
30	Phenyl salicylate	-	23.952	RI, MS	0.599 ^a^	-	-	-	-

Ref. is the reference of odor description in same row. Numbers in a row that does not share the same alphabetical letter represent significant differences (*p* < 0.05).

**Table 2:** Descriptive sensory analysis result on aroma of four mulberries treated in different temperatures.

Lexicon	Definition/Reference	Sample 1	Sample 2	Sample 3	Sample 4
Cucumber	Aromatics associated with cucumber (Ref. Freshly cut cucumber)	2.50 ^a^	1.55 ^b^	0.00 ^c^	0.00 ^c^
Green/grassy	Aromatics associated with grass (Ref. The rind of a watermelon)	0.87 ^a^	0.59 ^b^	0.00 ^c^	0.00 ^c^
Sweet aromatics	Aromatics associated with syrup (Ref: Oligosaccharides, CJ Cheiljedang, Seoul, Republic of Korea)	0.87 ^a^	0.80 ^a^	0.07 ^b^	0.00 ^b^
Raisin	Aromatics associated with raisin (Ref. Raisins, Sunview, CA, USA)	0.00 ^c^	0.00 ^c^	2.00 ^a^	1.48 ^b^
Sour aromatics	Aromatics associated with citric acid (Ref. Citric acid, Sigma-Aldrich, MO, USA)	0.00 ^b^	0.00 ^b^	0.47 ^a^	0.68 ^a^
Grape juice	Aromatics associated with grape juice (Ref. Jabez grape juice, Yeongdong Jabez Community, Yeongdong, Republic of Korea)	0.00 ^c^	0.00 ^c^	0.55 ^b^	1.00 ^a^
Dried seafood storage	Aromatics associated with Dried seafood storage (Ref. Woorim stock pack, Gijangfood, Busan, Republic of Korea)	0.00 ^b^	0.00 ^b^	0.70 ^a^	0.15 ^b^
Medicinal	Aromatics associated with hospital (Ref: Band-aid, Johnson-Johnson^®^ Korea, Seoul, Republic of Korea)	0.00 ^b^	0.87 ^a^	0.00 ^b^	0.00 ^b^

Numbers represent the mean value of aroma intensity of each sensory term, rated in a 15-pt Universal scale. Numbers in a row that does not share the same alphabetical letter represent significant differences (*p* < 0.05).
